# Concurrent Synthesis and Immobilization of Ag Nanoparticles over TiO_2_ via Plasma Reduction for Photocatalytic Treatment of Methyl Blue in Water

**DOI:** 10.3390/ma14206082

**Published:** 2021-10-14

**Authors:** Noor Ul Huda Altaf, Muhammad Yasin Naz, Shazia Shukrullah, Abdul Ghaffar, Muhammad Irfan, Dominik Walczak, Adam Głowacz, Mater H. Mahnashi, Saifur Rahman, Grzegorz Królczyk, Ali O. Alqarni, Usama Muhammad Niazi

**Affiliations:** 1 Department of Physics, University of Agriculture Faisalabad, Faisalabad 38040, Pakistan; noorulhuda100@yahoo.com (N.U.H.A.); chabdulghaffar@yahoo.com (A.G.); 2 Electrical Engineering Department, College of Engineering, Najran University Saudi Arabia, Najran 11001, Saudi Arabia; miditta@nu.edu.sa (M.I.); srrahman@nu.edu.sa (S.R.); 3 Faculty of Mechanical Engineering, Opole University of Technology, 45-758 Opole, Poland; d.walczak@po.edu.pl (D.W.); g.krolczyk@po.edu.pl (G.K.); 4 Department of Automatic Control and Robotics, Faculty of Electrical Engineering, Automatics, Computer Science and Biomedical Engineering, AGH University of Science and Technology, 30-059 Kraków, Poland; adglow@agh.edu.pl; 5 Department of Pharmaceutical Chemistry, College of Pharmacy, Najran University, Najran 11001, Saudi Arabia; matermaha@gmail.com (M.H.M.); aoqarni@gmail.com (A.O.A.); 6 Department of Mechanical Engineering Technology, National Skills University Islamabad, Islamabad 44000, Pakistan; ukniaxi@gmail.com

**Keywords:** photodegradation, semiconductor photocatalyst, Ag-coated TiO_2_, synthetic dyes, plasma reduction method

## Abstract

Pure TiO_2_ nanoparticles (TiO_2_NPs) were produced via the sol–gel method and then coated with silver nanoparticles (AgNPs) to reduce their optical band gap. The concurrent synthesis and immobilization of AgNPs over TiO_2_NPs was achieved through the interaction of an open-air argon plasma jet with a solution of silver nitrate/stabilizer/TiO_2_NPs. The one-pot plasma synthesis and coating of AgNPs over TiO_2_NPs is a more straightforward and environmentally friendly method than others. The plasma-produced Ag/TiO_2_ nanocomposites were characterized and tested for their photocatalytic potential by degrading different concentrations of methyl blue (MB) in water. The dye concentration, oxidant dose, catalyst dose, and reaction time were also optimized for MB degradation. XRD results revealed the formation of pure AgNPs, pure TiO_2_NPs, and Ag/TiO_2_ nanocomposites with an average grain size of 12.36 nm, 18.09 nm, and 15.66 nm, respectively. The immobilization of AgNPs over TiO_2_NPs was also checked by producing SEM and TEM images. The band gap of AgNPs, TiO_2_NPs, and Ag/TiO_2_ nanoparticles was measured about 2.58 eV, 3.36 eV, and 2.86 eV, respectively. The ultraviolet (UV) results of the nanocomposites were supportive of the degradation of synthetic dyes in the visible light spectrum. The AgNPs in the composite not only lowered the band gap but also obstructed the electron–hole recombinations. The Ag/TiO_2_ composite catalyst showed 90.9% degradation efficiency with a 5 ppm dye concentration after 120 min of light exposure.

## 1. Introduction

Contamination of water resources by the discharge of industrial effluents is a serious global issue. The main sources of water contamination are industrialization, civilization, agricultural operations, and other environmental and global changes. Due to its numerous adverse effects and carcinogenic nature, organic pollutant-based pollution is particularly damaging to living creatures [1]. Organic dyes are widely employed in a variety of sectors, including textiles, leather, and paper, and have negative consequences for human health and the environment, such as increased toxicity, unpleasant smells, chemical oxygen demand, and changes in water quality [2].

The known chemical, biological, and physical processes for the removal of colorants from wastewater include adsorption, precipitation, ultra-filtration, flocculation, and reverse osmosis, among others. These methods are non-destructive, merely transferring any contaminants into the sludge [3]. Among these techniques, adsorption and photocatalysis are the most prominent alternatives for the removal of harmful contaminants from water. However, most of the semiconductor-based photocatalyst materials employed for this purpose can only respond to ultraviolet (UV) radiation, which significantly restricts their application in the visible light spectrum. In the realm of photocatalysis, developing novel visible light-active photocatalysts with high quantum efficacy has piqued the interest of researchers. Photocatalysis has enormous potential for environmental purification under UV and visible light sources [4]. The absorption of light by semiconducting materials, such as titanium dioxide (TiO_2_), hematite (α-Fe_2_O_3_), tungsten oxide (WO_3_), tin oxide (SnO_2_), copper oxide (CuO), and nickel oxide (NiO), has attracted the attention of the research community working on photocatalysis. Among these, nanosized TiO_2_ is the most frequently used photocatalyst due to its non-toxic nature, low-cost, high surface adsorption affinity, and distinctive chemical, physical, and optical properties. However, these benefits are only manifested when TiO_2_ is applied under the ultraviolet region of the light spectrum, especially for photocatalytic dye degradation applications [5]. TiO_2_ is only active under UV radiation due to its high band gap (3.2–3.3 eV), which accounts for just 5% of the solar spectrum, while visible light accounts for 45% of solar spectrum [6]. Another drawback of using pure TiO_2_ is the fast recombination of photo-generated e^−^/h^+^ pairs and trapping in sub-surfaces [7].

The discrepancies discussed above can be addressed through the anion doping and metal ion doping of TiO_2_. Generally, silver (Ag), gold (Au), platinum (Pt), nickel oxide (NiO), graphene, and SnO_2_ are known to improve the photocatalytic activity of TiO_2_ in the visible region of the electromagnetic spectrum. As a result of these alterations, TiO_2_ can be used in a variety of applications [8]. Several methods are currently being employed to enhance the photoactivity of photocatalysts, such as forming a heterojunction by coupling TiO_2_ with another semiconductor material of low gap energy. A semiconductor dopant with low gap energy acts as a sensitizer. First, the dopant excites itself, then it causes TiO_2_ excitation by allowing photoelectrons to flow from its conduction band to that of the titanium dioxide [9,10,11]. Najafidoust et al. [12] produced TiO_2_ coatings with a biotemplate for the photodegradation of MB in wastewater. The titanium-coated agar showed high MB removal in just 45 min of illumination. Zhao and Chen [13] synthesized Ag/TiO_2_ using the sol–gel method. The size of the particles remained in the range of 1–2 nm. They used this nanocomposite for the removal of MB from water under ultraviolet radiation exposure. The photocatalyst was not very effective under visible light due to the very small particle size and the large band gap. Wei et al. [14] used a solvothermal process to produce Ag/TiO_2_ nanocomposites for the removal of organic pollutants crystal violet (CV) and rhodamine B (RhB). They reported a pollutant removal rate of 80–85% when the solution was exposed to visible light.

The reported methods for synthesizing Ag/TiO_2_ photocatalysts are difficult to conduct, time consuming, and require rigorous experimental conditions. These methods also contribute to environmental pollution, either directly or indirectly. The one-pot plasma synthesis and coating of AgNPs over TiO_2_NPs is a more straightforward and ecofriendly method compared to the other methods. However, the concurrent synthesis and immobilization of Ag nanoparticles over TiO_2_ via the plasma reduction method for the production of heterojunction photocatalysts is not widely reported in the published literature. In this study, pure AgNPs andTiO_2_NPs and Ag/TiO_2_ nanocomposites were produced for the purpose of dye degradation. The deposition of Ag particles onto TiO_2_ was achieved by means of a plasma–liquid interaction. The plasma reduction method was considered for the synthesis of the Ag/TiO_2_ nanocomposite, as it is one of the best know strategies or achieving controlled particle shape and size. Being simple, efficient, and ecofriendly, it can easily be scaled up to the largescale production of photocatalysts. The effect of the Ag/TiO_2_ photocatalyst, along with pure Ag and TiO_2_ nanoparticles, on the photodegradation of the synthetic dye was assessed under the visible light spectrum. Recycling experiments were also conducted to check the stability and practical value of the photocatalyst.

## 2. Experimental Section

### 2.1. Materials

Analytical-grade chemical reagents were supplied by Sigma-Aldrich (St. Louis, MO, USA). Titanium (IV) isopropoxide, ethanol, silver nitrate, and sodium hydroxide were used in the synthesis of pure Ag and TiO_2_ nanoparticles and Ag/TiO_2_ nanocomposites. The chemical reagents were used as supplied without undergoing any additional treatment.

### 2.2. Synthesis of Pure AgNPs

**Figure 1:**
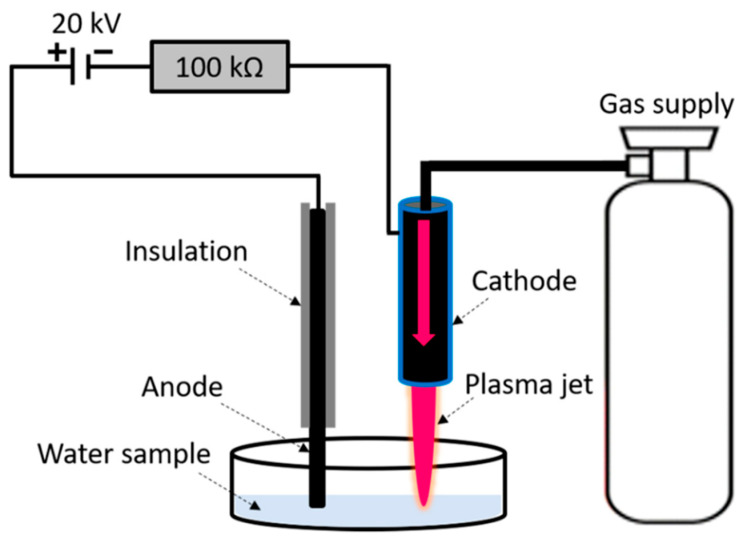
An argon plasma jet setup for production of nanoparticles by reduction the metal ions.

Applying a conventional one-step method, a 100 mL aqueous solution was produced by mixing 5 mM AgNO_3_ and 1 mM sucrose powder in deionized water. The solution of AgNO_3_ and sucrose was placed under an in-house built plasma jet. A detailed diagram of the plasma jet is given in Figure 1 [15]. The setup consisted of a DC voltage supply (1–20 kV), plasma jet, graphite electrode, argon gas supply, and resistance box. The input voltage was varied to sustain a stable plasma jet of argon gas. The interaction between the plasma jet and the solution was facilitated in an open atmosphere. The jet itself worked as a cathode when a graphite rod was connected to the positive terminal of the battery. A suitable resistance from the resistance box was selected to avoid short-circuiting and breakdown during plasma exposure. The plasma jet consisted of high reactive species, photons, excited species and other radicals. The H-radical is thought to be most reactive species during liquid–plasma interaction. It is also the major reducing agent in acidic solutions. The production of AgNPs by electron and H radicals’ irradiation under a plasma jet was initiated due to a fast coefficient rate (Equations (1) and (2)) [16]. Plasma’s active H^+^ radicals and electrons reduce Ag cations to AgNPs when they interact with an aqueous solution. Within 30 min of reaction time, the solution changes from transparent to black, indicating that AgNO_3_ has been reduced to AgNPs. The resulting AgNPs were washed multiple times. The nanoparticles were given heat treatment for 2 h in a vacuum oven at 80 °C.

(1) $${\mathrm{Ag}}^{+}+e_{\mathrm{aq}}^{-}\to \mathrm{Ag}$$

(2) $${\mathrm{Ag}}^{+}+H\to \mathrm{Ag}+H^{+}$$

### 2.3. Synthesis of TiO_2_NPs

The sol–gel method was adopted to synthesize TiO_2_NPs, rather than the plasma liquid interaction (PLIs) process, due to the very fast hydrolysis of its precursor (TTIP) at room temperature. Also, the sample may burn during plasma exposure and damage the apparatus due to direct interaction of the plasma with the solution. The sol–gel process was used to synthesize TiO_2_NPs using HNO_3_. First, 9 mL of titanium tetra isopropoxide was added to 25 mL ethanol and stirred for 1 h. Then 200 mL of deionized water was mixed with 2 mL of HNO_3_. This aqueous solution was then injected into a combination of ethanol and TTIP, drop by drop. The mixture was stirred to convert it into a gel at 60 °C. The obtained gel was dried at 100 °C in an open atmosphere to obtain nanocrystalline titanium dioxide. After annealing for 2 h at 400 °C, crystalline TiO_2_NPs were obtained. The obtained nanocrystals were ground to obtain a fine powder of TiO_2_NPs.

### 2.4. Synthesis of Ag/TiO_2_ Nanocomposite

A one-step deposition process was used to produce the Ag/TiO_2_ catalyst, as illustrated in Figure 1. Pure TiO_2_ was immersed in a solution of AgNO_3_ at a certain concentration (0.3 g). TiO_2_ was dispersed in a 2 mM AgNO_3_ solution prepared with 50 mL of water and ethylene glycol in a 9:1 ratio. About 0.1 M NaOH was also added dropwise into the solution with continuous stirring to enhance the dispersion of the AgNPs. The mixture was treated with an argon plasma jet (Figure 1) [17]. The plasma treatment time was limited to 30 min. After that, the mixture was filtered, rinsed with water, and dried for 12 h at room temperature. The whole plasma-assisted synthesis process can be explained by the following reactions:

(3) $${\mathrm{AgNO}}_{3}+\mathrm{NaOH}\to \mathrm{AgOH}+{\mathrm{NaNO}}_{3}$$

(4) $$2\mathrm{AgOH}\to {\mathrm{Ag}}_{2}O+H_{2}O$$

(5) $$e^{*}+\mathrm{Ar}\to {\mathrm{Ar}}^{*}+e$$

(6) $$e^{*}+H_{2}\to H_{2}{}^{*}+e$$

(7) $$2{\mathrm{Ag}}^{+}+H_{2}{}^{*}+2e\to 2\mathrm{Ag}+H_{2}$$

### 2.5. Characterizations

FTIR (Spectrum Two, Perkin Elmer, Waltham, MA, USA) analysis was employed to identify the functional groups of the pure AgNPs, pure TiO_2_NPs, and Ag/TiO_2_ nanocomposite. X-ray diffractometry (D8, Bruker, Billerica, MA, USA), CuK_α_: 0.15046 nm, V: 40 kV, I: 100 mA, scan rate: 2° min^−1^) was employed to study the crystalline structures of the pure and composite nanoparticles. X-ray diffraction patterns were produced over a 2θ range of 10–80°. The surface morphology and immobilization of AgNPs on TiO_2_NPs was assessed by generating SEM micrographs (Nova Nano SEM 450, FEI, Hillsboro, OR, USA) and TEM micrographs (JEM-2100 F, JEOL, Tokyo, Japan). The chemical composition of the nanocomposite photocatalyst was checked through EDX (Nova Nano SEM 450, FEI, Hillsboro, OR, USA) analysis. Absorbance spectra were recorded using a UV spectrometer (Lambda 950, PerkinElmer, Waltham, MA, USA) with a wavelength range of 200–800 nm to study the band gap of the pure and composite nanoparticles. The UV–Vis absorption spectra of MB solutions after irradiation were recorded using deionized water as a reference sample.

### 2.6. Photocatalytic Degradation Tests

The activity of Ag/TiO_2_ was ascertained by degrading MB dye under simulated visible light. The photocatalytic activity of pure AgNPs can be improved by modifying them under visible light and ultraviolet light. The initial absorption edge of pure TiO_2_ NPs is calculated as 380 nm, which shows that TiO_2_ can only be activated by UV light. The comparison of the absorption edges of Ag/TiO_2_ nanocomposite under different dye loading amounts with those of pure TiO_2_NPs reveals that the absorption edge of Ag/TiO_2_ nanocomposite under different dye loading amounts shows a slight red shift, which indicates enhanced photodegradation in the visible region of the light spectrum.

The stock solution of crystal MB was formed by dissolving 1 g of dye in 100 mL of distilled water. Then, a 1 mL solution of MB dye from the stock solution was added into another 100 mL of distilled water. About 0.1 g of Ag/TiO_2_ catalyst and varying amounts of oxidant were dissolved into the above solution by stirring it for few minutes in an ultrasonicator (S-DS-3, Stalwart Instruments, Boca Raton, FL, USA). The reaction mixtures of the desired concentrations (5, 10, and 20 ppm) were formed and hydrogen peroxide (H_2_O_2_) was added as an oxidizer. Before conducting the photocatalytic reactions, the solutions were held in dark conditions to ensure adsorption–desorption equilibrium. The photodegradation of MB was performed in a wooden box. A 700 W light source (Philips HPL n 700W/542 E40, Tokyo, Japan) was used as a visible light source. The solutions were then illuminated with simulated visible light irradiation for 120 min. During irradiation, a 3 mL solution was taken every 20 min and centrifuged at 2000 rpm for 20 min to remove the photocatalyst materials from the solution. UV–Vis absorption spectroscopy (CE 7200, Cecil Instruments, Cambridge, UK) was used to determine the residual amount of MB dye after these regular intervals of time. The dye degradation was studied by varying the parameters, such as the pH of the medium, dye concentration, oxidant dose and irradiation time. The photodegradation efficiency of each sample was calculated using the formula:

(8) $$\eta =\frac{\left(A_{0}-A_{t}\right)}{A_{0}}\times 100$$

where *A_t_* is initial absorbance and *A_t_* is final absorbance of dye after time (*t*). The degradation efficiency of the reaction is represented by *η*.

To ascertain the stability and reusability of the Ag/TiO_2_ nanocomposite during dye degradation experiments, five cycles of dye degradation were conducted with same nanocomposite. The degradation percentage after each photocatalytic cycle was measured to quantify the loss in degradation and, thus, the reusability of the nanocomposite. After each degradation cycle, the nanocomposite was filtered, rinsed, and dried to make it ready for next cycle.

## 3. Results and Discussion

### 3.1. FTIR Analysis

**Figure 2:**
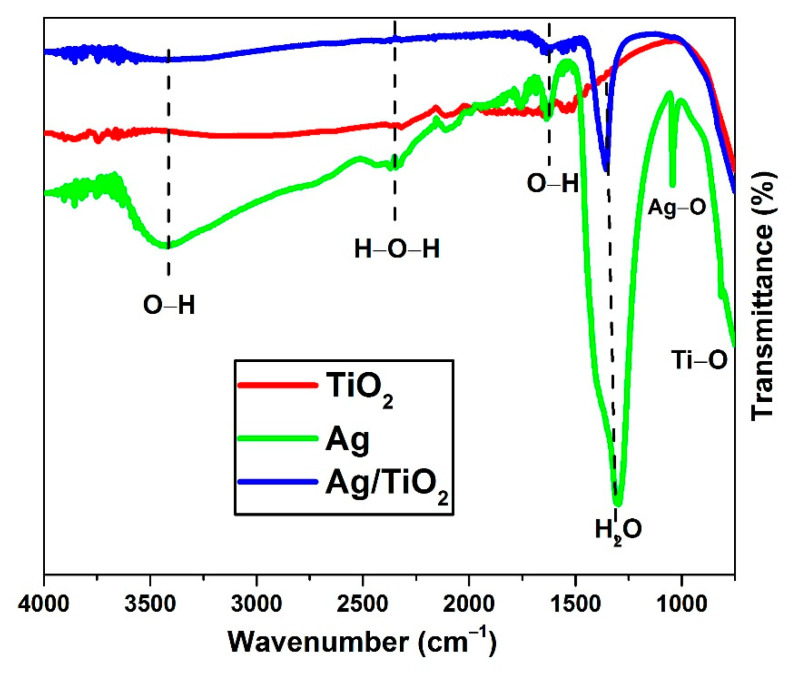
FTIR analysis of pure and composite nanoparticles.

The surface chemistry and formation of oxygen functional groups on the surface of pure Ag, TiO_2_NPs, and Ag/TiO_2_ nanocomposite were investigated using FTIR analysis. Figure 2 depicts the infrared spectra of the synthesized samples. A broad band at 448 cm^−1^ is referred to the Ag–O stretching mode. The absorption band at 758 cm^−1^ is assigned to TiO_2_ lattice vibration, as can be observed from the spectra (Ti–O stretching mode). O–H stretching of hydroxyl groups is represented by the peaks at 3135 cm^−1^. A broad band in the range of 2900–3420 cm^−1^ is associated with the H–O–H bending vibrations of water adsorbed on the surface of the nanocomposite [18]. The presence of hydroxyl groups enhances photocatalytic activity because OH groups act as a major scavenger of photo-generated electrons and holes, resulting in the generation of hydroxyl radicals (OH), which are essential for the breakdown of MB dye. Transmittance peaks, spotted at 474–750 cm^−1^, are associated with OH vibrations. These vibrations come into play due to H–O–H interactions between AgNPs and TiO_2_NPs and hydrogen bonding [19]. The functional groups of nanoparticles extracted via the FTIR analysis are reported in Table 1.

### 3.2. Structural Analysis

**Figure 3:**
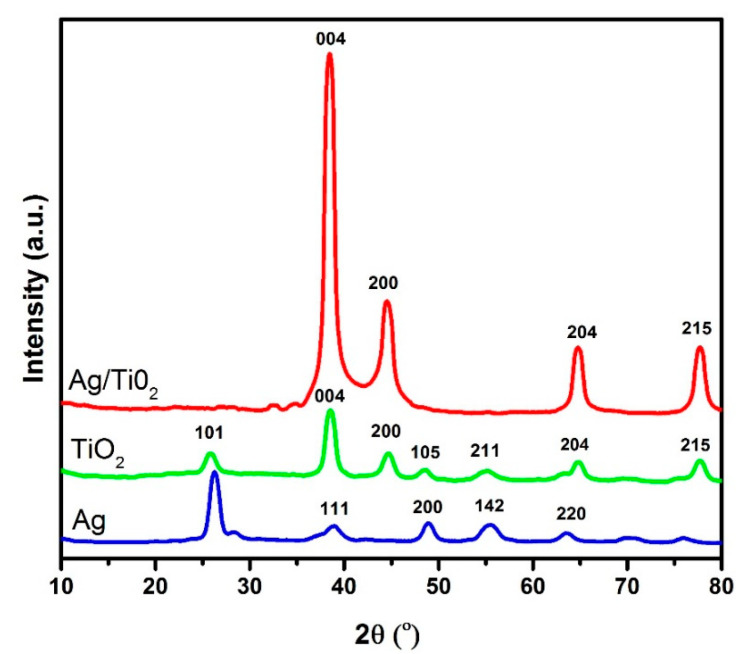
XRD spectra of pure and composite nanoparticles.

Figure 3 illustrates the diffraction spectra of AgNPs, TiO_2_NPs, and Ag/TiO_2_ nanocomposites. The widening and noise in XRD peaks was most likely due to the presence of macromolecules in the sample. Using the Scherrer formula, the grain size was determined from the major diffraction peaks of the plane (111). The Scherrer formula is given by the equation:

(9) $$D_{p}=\frac{k\lambda }{\beta_{1/2}cos\theta }$$

where, *β* is the diffraction broadening (FWHM), *λ* is X-ray wavelength, *k* is a constant (0.94), and *θ* is the Bragg’s angle. The obtained average grain sizes of pure Ag and TiO_2_ were about 12.36 nm and 18.09 nm, respectively. The grain size of Ag/TiO_2_ composite was about 15.66 nm. The XRD peaks at 2*θ* of 38.541°, 44.637°, 64.858°, and 77.742° were ascribed to (004), (200), (204), and (215), respectively. Furthermore, the Ag/TiO_2_ pattern showed a unique peak of Ag at 2*θ* of 38.54°. This demonstrates that AgNPs bonded to TiO_2_NPs and did not influence the crystal lattice of the TiO_2_ [20]. The structural parameters, extracted from XRD spectra, are reported in Table 2.

### 3.3. SEM/TEM Analysis

**Figure 4:**
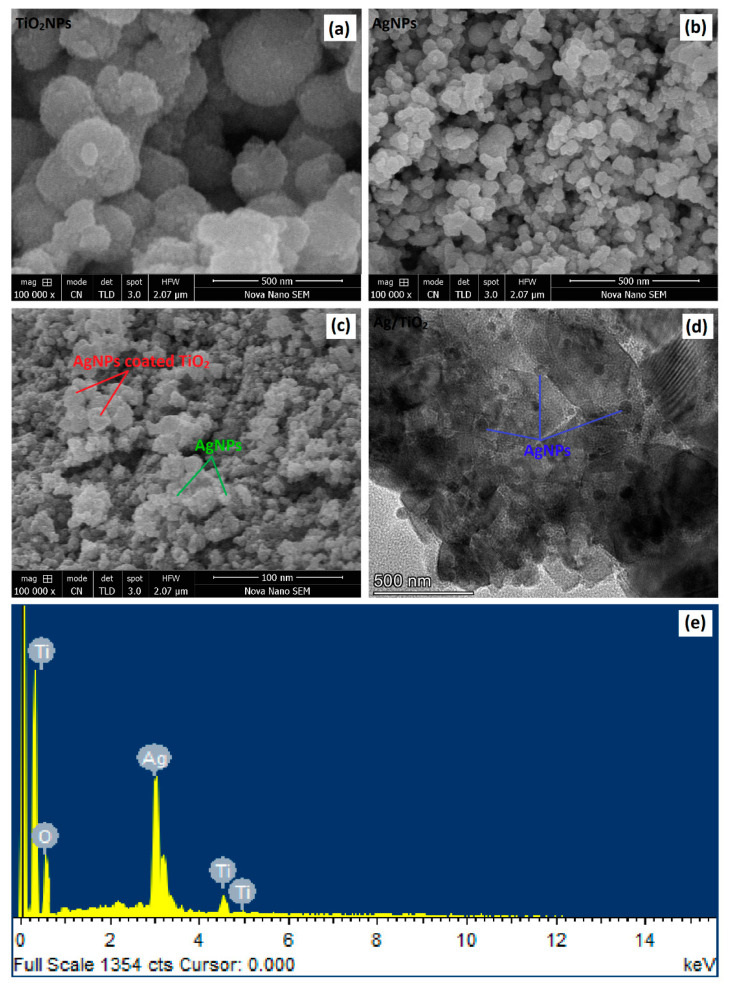
SEM micrograph of pure TiO_2_NPs (**a**); SEM micrograph of pure AgNPs (**b**); SEM micrograph of Ag/TiO_2_ nanocomposite (**c**); TEM micrograph of Ag/TiO_2_ nanocomposite (**d**); and EDX spectrum of Ag/TiO_2_ nanocomposite (**e**).

The SEM morphology of AgNPs, TiO_2_NPs, and Ag/TiO_2_ NC are depicted in Figure 4. The silver nanoparticles grew in the form of nanoclusters. Some agglomeration was also observed in the SEM images. The TiO_2_ nanoparticles were more spherical and dispersed compared to the AgNPs. However, it is worth mentioning here that the AgNPs were synthesized using the PLI process, whereas the TiO_2_NPs were produced via the facile sol–gel method. The difference in shapes of these nanoparticles might be due to the difference in synthesis methods, which involve different procedures and reactions. The particle sizes of AgNPs and TiO_2_NPs were found to be about 24.72 nm and 30.27 nm, respectively. A uniform distribution of particle size in AgNPs and TiO_2_NPs was observed in the SEM images.

Figure 4 shows AgNPs immobilized over TiO_2_ nanoparticles. The PLI method was used for the concurrent synthesis and deposition of AgNPs over TiO_2_. When Ag^+^ was deposited on TiO_2_, the Ag^+^ reduced to Ag^0^ and finally oxidized to AgNPs. The size of Ag/TiO_2_ particles was slightly smaller than that of TiO_2_NPs. The deposition of AgNPs onto TiO_2_NPs was also checked through TEM. The TEM micrographs in Figure 4 show the attachment of Ag to TiO_2_, labelled using blue and red marks respectively. The EDX spectrum of Ag/TiO_2_ also confirms the presence of silver, titanium, and oxygen in the composition. No extra peaks were observed in the EDX spectrum, which confirms the formation of pure phases of Ag and TiO_2_ nanoparticles.

As shown in Table 3, BET analysis revealed the average surface area of pure AgNPs, pure TiO_2_NPs, and Ag/TiO_2_ nanocomposite to be 4.3, 4.9, and 4.7 m^2^/g, respectively. These calculations were made by assuming that the particles were solid spheres with a smooth surface morphology. The BET analysis of the prepared nanomaterials agreed well with the SEM analysis. Their pore volumes were determined to be approximately 0.0641, 0.0803, and 0.0695, respectively. The pure TiO_2_NPs exhibited the largest surface area and pore volume, followed by the Ag/TiO_2_ nanocomposite, and pure AgNPs. The surface porosity may vary with particle size and method of synthesis. It is important to mention here that catalytic activity is mainly driven by the surface area. The larger the particle surface area, the higher the photocatalytic activity of the photocatalyst will be. However, this assumption is only valid when the photocatalyst’s band gap energy is reasonable low and the rate of electron–hole recombination is limited.

### 3.4. Optical Band Gap

**Figure 5:**
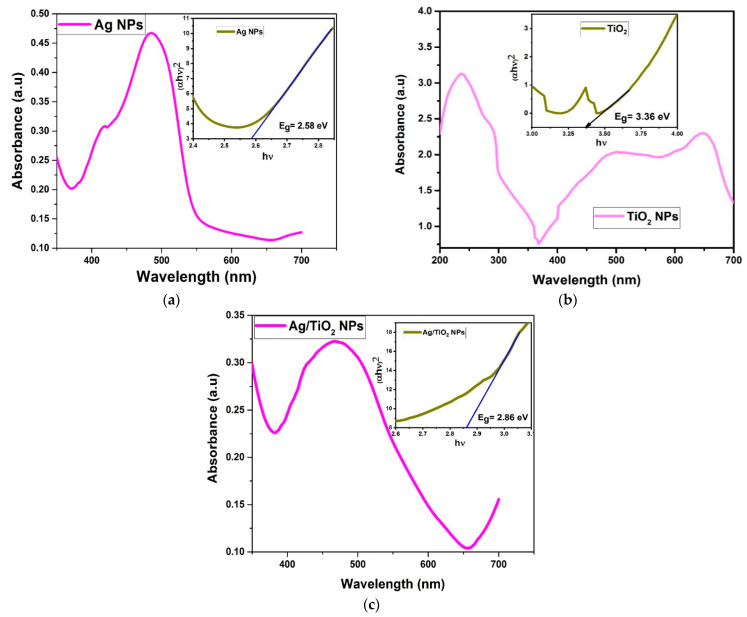
UV absorption spectra and band gap energy measurements of pure (**a**) AgNPs, (**b**) pure TiO_2_NPs and (**c**) Ag/TiO_2_ nanocomposite.

The UV–Vis absorption spectra of pure AgNPs, pure TiO_2_NPs, and the Ag/TiO_2_ nanocomposite are shown in Figure 5a–c. The absorption edges of these nanoparticles were measured at 484 nm, 236 nm, and 468 nm, respectively. Using the Tauc plot equation, the band gap energy of the synthesized materials can be expressed as:

(10) $$\alpha h\upsilon =B{\left(h\upsilon -Eg\right)}^{n}$$

The coefficient of absorption, Planck’s constant, band gap energy, light frequency, and a constant are represented by α, h, υ, and E, respectively. The energy band gaps of AgNPs, TiO_2_NPs, and Ag/TiO_2_ composites were calculated as 2.58 eV, 3.36 eV, and 2.86 eV, respectively. The band gap of pure TiO_2_ reduced to 2.86 eV after the immobilization of AgNPs on its surface. The conduction band shifted downward and the valence band shifted slightly upward. Also, the absorption edge of TiO_2_NPs extended to the visible light range in the presence of AgNPs [21]. Both factors are vital for improved photocatalytic activity of the Ag/TiO_2_ nanocomposite.

### 3.5. Mechanism of Photodegradation of Methyl Blue

**Figure 6:**
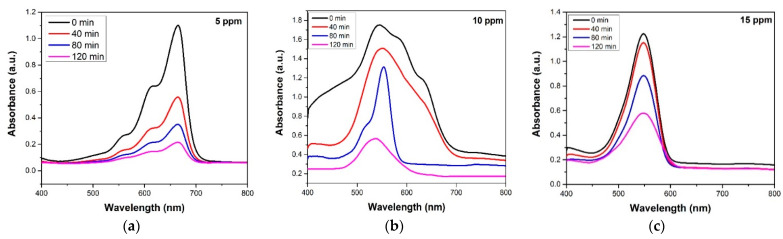
UV absorption spectrum of (**a**) 5 ppm, (**b**) 10 ppm, and (**c**) 15 ppm solutions of MB after different degradation times.

The activity of AgNPs, synthesized using the PLI technique, has been reported in our previous work [17]. AgNPs have been synthesized under similar conditions and tested for their ability to decompose MB molecules. The activity of TiO_2_NPs produced via the sol–gel method has been reported by Zulmajdi et al. [22]. So, rather than focusing on the photocatalytic activity of pure AgNPs and TiO_2_NPs, the present study deals with the degradation of MB using Ag/TiO_2_ nanocomposite as a catalyst. Using this photocatalyst, varying concentrations of MB in water were degraded. The results are presented in Figure 6a–c.

**Figure 7:**
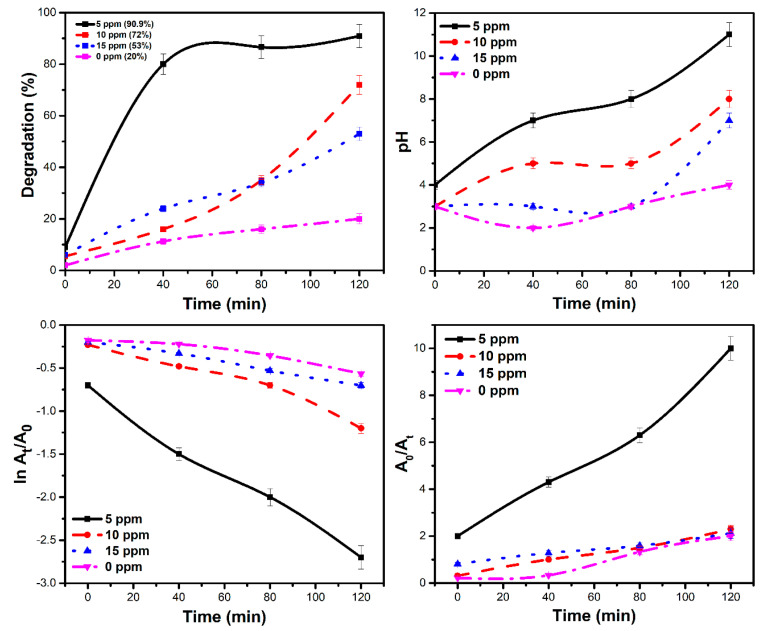
Photolysis and catalytic dye degradation efficiency profiles for different pH values, pseudo-1st-order and 2nd-order models.

The photodegradation efficiency of Ag/TiO_2_ composite under simulated light irradiation was 90.9%, 72% and 53% for 5 ppm, 10 ppm, and 15 ppm solutions, respectively, after 120 min of irradiation, as shown in Figure 7. The use of an Ag/TiO_2_ composite has a number of benefits. The silver nanoparticles have a narrow band gap, which, when combined with TiO_2_, reduces the band gap of Ag/TiO_2_. The reduced band gap allows the composite to perform well even under visible light irritation.

**Figure 8:**
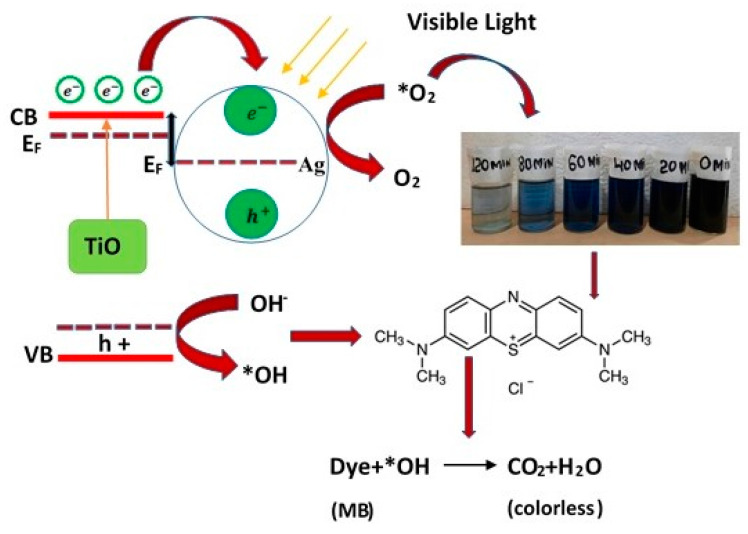
Schematic of mechanism of photoactivation of Ag/TiO_2_ catalyst for degradation of synthetic dyes.

The large band gap of TiO_2_ limits e–h pair formation under visible light excitation. The AgNPs in the composite work as photosensitizers. They harvest energy from the electromagnetic spectrum in the visible range and inject the excited electrons into the conduction band of TiO_2_. These electrons then become available for photocatalytic degradation reactions. Figure 8 depicts transfer process the generated electrons undergo in Ag/TiO_2_ when exposed to visible light. The electron–hole pairs produced by this process split efficiently under these conditions and can then contribute to photocatalytic degradation reactions. As a result, Ag/TiO_2_ shows maximum photocatalytic activity in the visible part of the spectrum [4].

The mechanism proposed for the removal of synthetic dyes using an Ag/TiO_2_ photocatalyst is illustrated in Figure 8 [23,24]. This mechanism is proposed for visible light irradiation of the photocatalyst. The enhanced photocatalytic activity of the Ag/TiO_2_ catalyst was proposed based on the surface plasmon resonance of AgNPs and the reduced band gap of the binary nanocomposite. Table 4 compares the efficiency of dye degradation using different metal catalysts and procedures. The mechanism of this study consists of 3 steps: (1) e^−^/h^+^ pair generation and transfer, (2) reactive radical and superoxide generation, and (3) decomposition of target molecules. The MB dye can be oxidized by common reactive species, such as superoxide, hydroxyl radicals and h^+^ during photocatalytic activity. These intermediates and species react with the final product, causing them to disintegrate into mineralized products, as indicated in Equation (15). The Ag cations change the catalytic activity of TiO_2_ by trapping electron holes and modifying the h^+^/ e^−^ pair recombination rate through the reactions given below. Table 4 summarizes the photocatalytic activity of Ag and TiO_2_NPs based on various parameters, including methodology, type of dye, irradiation time, and light source.

(11) $$\mathrm{Ag}/{\mathrm{TiO}}_{2}+\;h\upsilon \to {\;e}_{\mathrm{CB}}^{-}+h_{\mathrm{VB}}^{+}$$

(12) $$e_{\mathrm{CB}}^{-}+O_{2}\to O_{2}^{-}$$

(13) $$O_{2}^{-}+H_{2}O\to {\;\mathrm{OH}}^{-}+H_{2}O$$

(14) $${\mathrm{OH}}^{-}+h_{\mathrm{VB}}^{+}\to \mathrm{OH}$$

(15) OH+organic pollutants→Photodegradation products

### 3.6. Effect of pH on Dye Degradation

It was observed that the pH of the solution had a significant impact on the photocatalytic degradation of pollutants. To check the role of pH in dye degradation, tests were conducted with different pH values (3–11) in 1.0 g/L of Ag/TiO_2_ and a 3 mM H_2_O_2_ oxidant dose. The dye concentrations were 5 ppm, 10 ppm, and 15 ppm. The solutions were sonicated to achieve a good dispersion of the catalyst. Titanium oxide’s surface charge changes when the pH of the solution changes, affecting the catalytic activity of TiO_2_ particles. In acidic and alkaline media, the surface of TiO_2_ becomes positive and negative, respectively. Furthermore, in acidic or alkaline media, the TiO_2_NPs surface can protonate or deprotonate, as seen in the reaction given below.

(16) $$\mathrm{TiOH}+H^{+}\to {\mathrm{TiOH}}_{2}^{+}$$

(17) $$\mathrm{TiOH}+{\mathrm{OH}}^{-}\to {\mathrm{TiO}}^{-}+H_{2}$$

The results presented in Figure 7 indicate a maximum degradation efficiency at pH 8, as indicated by a lower dye concentration. In an alkaline solution, the adsorbed OH* species react with photogenerated holes to form OH* radicals that are resistant to dye degradation. More OH^−^ ions are accessible on the surface to be oxidized in an alkaline medium, resulting in increased photocatalytic activity. Similar findings have been observed by other researchers [30].

### 3.7. Catalyst and Oxidant Dose

The concentration of H_2_O_2_ was changed in solution to see how this affects the dye degradation process. The oxidant concentration variations were 1, 3, and 5 mM. The rate of degradation increased in the presence of the oxidant due to the fast decomposition of hydrogen peroxide into OH radicals, as depicted in Equation (18).

(18) $$H_{2}O_{2}+O_{2}^{*}\to {\mathrm{OH}}^{*}+{\mathrm{OH}}^{-}+O_{2}$$

The results show that increasing the amount of oxidant influenced the dye degradation. The hydroxyl radical (OH*) plays a significant role in photocatalytic breakdown of MB by acting as an oxidant and consuming perhydroxic acid. The maximum degradation was achieved with a 1 mM dosage of oxidant. Separate degradation tests were performed with initial dye concentrations of 5, 10, and 15 ppm to quantify the impact of initial dye concentration on the reaction rate. All degradation tests were performed using 1.0 g of Ag/TiO_2_ loading. After 120 min of reaction, about 90.9%, 72% and 53% dye degradation was possible, with initial dye concentrations of 5, 10 and 15 ppm, respectively. With increasing dye concentrations, the rate constant (*k*) value decreased linearly from 20.05 × 10^−3^ to 6.1 × 10^−3^ min^−1^ for the pseudo-1st-order model and from 66.6 × 10^−3^ to 10.9 × 10^−3^ min^−1^ for the pseudo-2nd-order model. The data in Table 5 reveal a decrease in rate constant with a rise in initial dye concentration. The rate of the reaction, on the other hand, was calculated using Equation (19).

(19) $$lnA=kt+lnA_{o}$$

The amount of available active sites on the nanocatalyst decreased as the initial dye concentration and time interval increased, resulting in a decrease in the rate constant. According to the data in Table 4, the degradation of dye decreased from 90.9% to 53% when the initial dye concentration was increased from 5 ppm to 15 ppm.

### 3.8. Catalyst Stability

**Figure 9:**
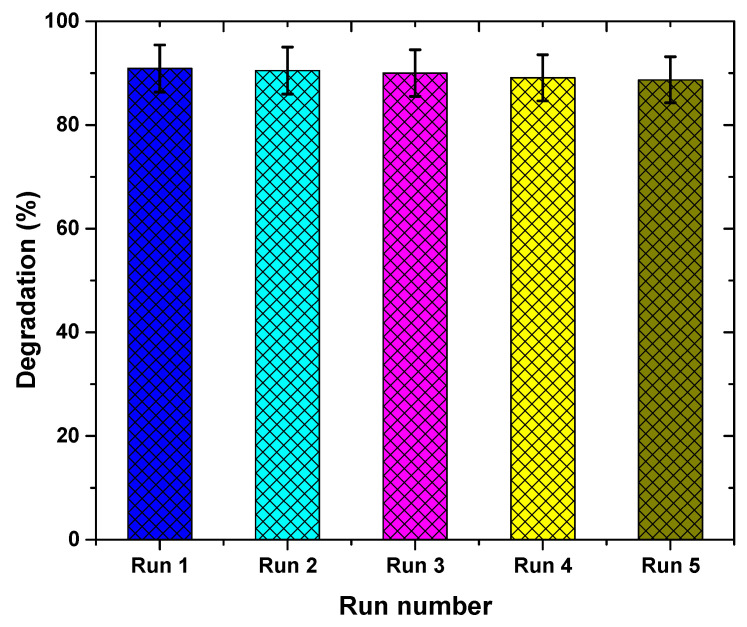
Reusability test of Ag/TiO_2_ over 5 degradation cycles with 5 ppm dye concentration.

**Figure 10:**
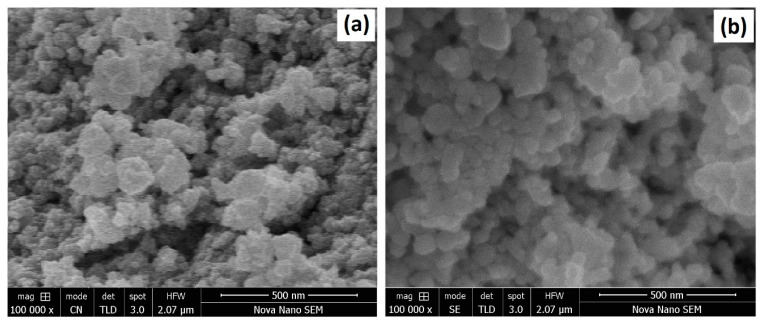
SEM images of the photocatalyst (**a**) before performing dye degradation and (**b**) after performing five dye degradation cycles.

The stability of the Ag/TiO_2_ composite catalyst was evaluated by using the same catalyst in repeated dye degradation cycles. The degradation process was repeated for five runs and the results are reported in Figure 9. The tested photocatalyst proved highly stable over repeated cycles. Reusability tests of Ag/TiO_2_ over five degradation cycles were conducted with a dye concentration of 5 ppm. A degradation efficiency of approximately 90.9% was achieved after first cycle of 120 min. The tested catalyst was then washed and dried for the next dye removal cycle. The catalyst lost only 2% in degradation efficiency after performing five degradation cycles. These findings confirm that Ag/TiO_2_ nanocomposite has good stability and reusability. Figure 10 shows SEM images of the photocatalyst before and after performing the five dye degradation cycles. The shape of the particles was more defined after recycling the photocatalyst than when it was in pristine condition. Some of the AgNPs that are only loosely bonded to TiO_2_NPs, as well as those agglomerated into clusters, detach and dissolve in the solution, leaving behind stable nanocomposite particles.

### 3.9. Practical Value

The reported data were extracted from laboratory experiments with known concentrations of a single organic pollutant. However, real environmental samples contain multiple organic and inorganic pollutants. Although the tested photocatalyst was found to be effective against organic pollutants, commenting on its effectiveness against inorganic pollutants would be difficult at this stage. In addition, specific mechanisms for practical applications of the tested photocatalyst in environmental pollution remediation are still only under development.

## 4. Conclusions

Pure TiO_2_ nanoparticles were produced via the sol–gel method and then exposed to an argon plasma jet while dissolved in a solution of silver nitrate. The plasma-reactive radicals reduced the silver ions into nanoparticles and facilitated their immobilization over TiO_2_ to form an Ag/TiO_2_ composite. The photocatalytic activity of the prepared composite was tested by using it to degrade methyl blue dye. The optical band gap energies of AgNPs, TiO_2_NPs, and the Ag/TiO_2_ composite were found to be approximately 2.58 eV, 3.36 eV, and 2.86 eV, respectively. The band gap of pure TiO_2_ was reduced to 2.86 eV after the immobilization of AgNPs on its surface. The OH and Ti–O–Ti vibrations were confirmed by means of a FTIR analysis of the composite. Adsorbed OH ions are important in photocatalysis because they trap carriers, resulting in reactive OH* radicals, which are the driving force behind the process. The Ag cations can change the catalytic activity of TiO_2_ by trapping electron holes and modifying h^+^/e^−^ pair recombination rate. After 120 min of irradiation, it was found that MB dye had degraded by 90.9%, 72% and 53% in solutions that contained initial MB concentration of 5, 10 and 15 ppm, respectively. The rate constant decreased linearly from 20.05 × 10^−3^ to 6.1 × 10^−3^ min^−1^ for the pseudo-1st-order model and from 66.6 × 10^−3^ to 10.9 × 10^−3^ min^−1^ for the pseudo-2nd-order model with an increase in dye concentration. A degradation efficiency of about 90% was possible after the first cycle of 120 min. The catalyst was centrifuged and dried for the next cycle. The catalyst lost only 2% efficiency after completing five degradation cycles. These findings confirm the stability and reusability of the Ag/TiO_2_ nanocomposite. Further research is suggested to examine the effect of multiple stabilizers on the production and immobilization of AgNPs on TiO_2_NPs. The effect of plasma parameters and gas type on the structures of the nanocomposites should also be considered in future research undertakings.

## Figures and Tables

**Table 1:** Functional groups of nanoparticles extracted from FTIR spectra.

Functional Groups	Bond	Observed Wave Number (cm^−1^)
Alcohol	O–H stretching	3412
C–O bond	H–O–H stretching	2353
=C–H	O–H bend	1623
Ester carbonyl	H–O–H bend	1287
C–O bond	Ag–O bend	1047
Aromatic compound	Ti–O–Ti stretching	758

**Table 2:** The grain size, strain, and band gap of pure and composite nanoparticles.

Sample	Calcination Temp	Average Grain Size (nm)	Strain (ε)	Band Gap (eV)
AgNPs	0	12.36	0.42	2.58
TiO_2_NPs	350	18.09	0.46	3.36
Ag/TiO_2_NC	450	15.66	0.36	2.86

**Table 3:** Average surface area and pore volume of pure AgNPs, pure TiO_2_NPs and Ag/ TiO_2_ nanocomposites.

Sample	Nanoparticle Size (nm)	Surface Area (m^2^/g)	Pore Volume (cm^3^/g)
AgNPs	12.36	4.3	0.0641
TiO_2_NPs	18.09	4.9	0.0803
Ag/TiO_2_NC	15.66	4.7	0.0695

**Table 4:** A comparison of photocatalytic activity of LPI synthesized Ag/TiO_2_ composite with previous studies.

Sr. No	Method	Photocatalysts	Dye	Time (min)	Light Source	Ref.
1	Plasma Interaction Method	Ag/TiO_2_	MB	80	Visible light	Present Study
2	Facile sol–gel	TiO_2_, Pd/TiO_2_	MB, MO	120	UV-irradiation	[25]
3	Sol–gel	Zn/TiO_2_	RhB	-	Visible light	[26]
4	Co-precipitation	Ag_3_PO_4_/N-TiO_2_	RhB	120	Visible light	[27]
5	Green synthesis	Ag/TiO_2_	RhB, MB, MO	180	LED bulb	[28]
6	Solvothermal route	Ag@TiO_2_ Nanorods	RhB	80	Visible light	[29]

**Table 5:** Effect of pH, oxidant dose, dye concentration, and time on dye degradation efficiency.

Dye Conc. (ppm)	Oxidant Dose (mM)	Catalyst Loading (g/L)	pH	Degradation Efficiency (%)	K_1_	K_2_	R_1_^2^	R_2_^2^	Rate Constant Min^−1^ 1st Order	Rate Constant Min^−1^ 2nd Order
5	1	1.0	8	90.9	0.0032	0.013	0.99	0.98	20.05 × 10^−3^	66.6 × 10^−3^
10	3	1.0	5	72	0.00078	0.00163	0.96	0.992	10.06 × 10^−3^	16.65 × 10^−3^
15	5	1.0	3	53	0.0002	0.00070	0.9941	0.991	6.1 × 10^−3^	10.9 × 10^−3^

## Data Availability

Data sharing is not applicable for this article.
